# Bioinformatic Approaches for Fungal Omics

**DOI:** 10.1155/2017/7270485

**Published:** 2017-03-15

**Authors:** Guohua Xiao, Xinyu Zhang, Qiang Gao

**Affiliations:** ^1^School of Computer Science, Fudan University, Shanghai 200433, China; ^2^Department of Psychiatry, Yale Medical School, Yale University, New Haven, CT 06511, USA; ^3^Department of Pharmacology, University of Texas, Southwestern Medical Center, Dallas, TX, USA

Fungal omics (including genomics, transcriptomics, metabolomics, proteomics, and lipidomics) are now broadly applied to help understand both the basic fungal biology and associated applications. Due to the advantages of the rapid development of Next-Generation Sequencing (NGS) technologies, bioinformatics algorithms, and the relatively smaller sizes of fungal genomes compared with other eukaryotes, sequencing and analysis of fungal genomics become much easier. Along with the acquisition of fungal genomic data, other omic data, such as proteomic data, have been increasingly reported.

Therefore, appropriate data mining of these omic data in depth and the obtained information can benefit our understanding of the complex fungal biological processes from genotype and physiology to phenotype, including cell-cell (microbial) communications and pathogen-host interactions and beyond. The research papers published in this special issue represent recent progress in the aspects, including RNA sequencing analysis, comparative proteomics, metagenomics, developments of new computational workflows, and fungal physiology and biology revealed by bioinformatic approaches. All of these papers provide novel ideas and technologies in the field and stimulate future research for fungal omics.

Xyr1 is one of the main transcription activators of (hemi)cellulases in the well-known cellulase producer* Trichoderma reesei*. L. Ma et al. identified the genes regulated by Xyr1 through RNA sequencing. Their results and analysis might help elucidate the regulation system for synthesis and secretion of (hemi)cellulases in* T. reesei*.

L. H. Tang et al. performed a comparative proteomic analysis of mycelial brown film formation in* Lentinula edodes*. These results provided useful help for future detailed investigations of the proteins linked to brown film formation.

J. Tang et al. analyzed the bacterial communities of two soy sauce aroma liquors. They provided new insights into the bacterial composition of the Chinese liquor Daqu and the fermentation process using high-throughput sequencing technology. At last, 17 phyla species were obtained from the Daqu samples, which were considered to be useful in the manufacture of Chinese liquors.

M. Převorovský et al. developed a computational workflow for the calculation of genome-wide splicing efficiency in* Saccharomyces cerevisiae* using strand-specific RNA-seq data. They demonstrated the functionality of the workflow using RNA-seq datasets from three spliceosome mutants and provided all relevant scripts in a ready-to-use form.

Y. Wang et al. compared the putative pathogenicity-related genes identified by T-DNA insertional mutagenesis (comprising 1024 genes) with the genes by microarray expression profiling (comprising 236 genes) in* Magnaporthe oryzae*. Only 13 genes were overlapped between the two gene lists. Their results of gene knockout mutants were also negative.

D. Lü et al. applied molecular cloning in combination with bioinformatic analysis to explore the function of* Bombyx mori* Lebocin 5 Gene. Their results showed that* Bombyx mori* Lebocin 5 Gene might play an important role in the immune response of silkworm to defend* B. bassiana* infection.

The guest editors are grateful to all authors and reviewers for their contributions to this special issue.

*Guohua Xiao* *Xinyu Zhang* *Qiang Gao*

